# A Seven-Decade Analysis: What Does the Gender Breakdown of Award Recipients in the All India Ophthalmological Society Reveal?

**DOI:** 10.7759/cureus.84438

**Published:** 2025-05-19

**Authors:** Sonia Phulke, Ashish Kumar, Priyanka Madaan, Amandeep Hans, Nidhi Malhotra

**Affiliations:** 1 Department of Ophthalmology, Dr. B. R. Ambedkar State Institute of Medical Sciences, Mohali, IND; 2 Department of Pediatric Medicine, Amrita School of Medicine, Faridabad, IND; 3 Department of Pediatric Neurology, Amrita School of Medicine, Faridabad, IND; 4 Department of Psychiatry, Dr. B. R. Ambedkar State Institute of Medical Sciences, Mohali, IND

**Keywords:** all india ophthalmological society, award, gender disparity, ophthalmology, women

## Abstract

Introduction

Professional awards and honors given by the medical societies help their members to boost their academic excellence and encourage leadership opportunities. The present study aimed to analyze the gender distribution of award recipients, identify any existing gender disparities, and assess factors influencing recognition in the field of ophthalmology.

Methods

This was an observational study with data extracted from the publicly available All India Ophthalmological Society (AIOS) website. Seventy-three years of data from the award recipients were reviewed. Individuals' gender was determined based on the first name and confirmed through internet searches of pronoun descriptors from professional websites. Key outcome measures were the gender distribution by year (1949-2022), category (achievement, scientific investigation/research, contribution to society/leadership, or lifetime achievement), and nomination type (self or society). Comparisons were made using Fisher's exact and chi-square tests when appropriate, with statistical significance set at a two-tailed P-value of <0.05.

Results

Of 452 award recipients across 31 AIOS award categories between 1949 and 2022, 323 recipients (71.5%) were men and 129 (28.5%) were women. Men received 96.7%, 78.6%, and 65.2% of awards in the contribution to society/leadership, achievement, and scientific investigation/research category, respectively, highlighting a significant underrepresentation of women in award distribution. Analysis of factors influencing gender distribution revealed that the award year, nomination type, and award category significantly impacted gender representation. Notably, fewer women were awarded before the start of the 21st century, and self-nominated candidates were predominantly skewed towards male recipients.

Conclusion

This study reveals significant gender disparities in award distribution within the Indian Ophthalmological Society. However, an increase in the proportion of female awardees over the years suggests a positive trend towards inclusivity.

## Introduction

The participation of women in all streams and facets of life is much needed to tap their potential and to promote equality. However, data suggest their underrepresentation in different walks of life, ranging from politics to academics [1-4]. In the 2024 global ranking, India ranks 129th in gender parity [5]. Although women in medical fields keep their flags high in research, publication, and mentoring students through a steady balance in their professional and personal lives, this does not limit the concerns about gender disparity and unequal opportunities for them. Furthermore, the underrepresentation of women has been observed in several medical societies. However, there is a relative lack of literature covering this issue across different medical streams in Asian countries.

With both medical and surgical aspects (including both primary and specialist eye care) being integral parts of ophthalmology, and many females opting for this specialty, the representation and recognition of females in this broad specialty can indirectly reflect the general trend in medical streams in a country. Western literature depicts the underrepresentation of women ophthalmologists in clinical practice, as well as in senior rank and leadership positions [6-8].

A few recent studies from Western countries have demonstrated a significant gender gap among award recipients of various ophthalmology societies, favoring men [9-11]. A similar gender gap is observed in the recipients of research funding [12,13]. Additionally, some studies report lower publication productivity among females across different sub-specialties of ophthalmology, including cornea and oculoplastics [12,14]. However, these aspects have not yet been evaluated in Indian ophthalmology societies.

Although there are significant social/cultural differences between India and Western countries, studies of Indian medical and surgical societies reveal similar or, in some cases, even worse levels of gender disparity. For instance, in the Association of Surgeons of India (ASI), as of November 2020, only 10% of members were women [15]. According to the All India Survey of Higher Education, among 17.05 lakh students enrolled in undergraduate medical sciences courses, 57.6% were women, and among the Ophthalmology postgraduates, 68.5% were women [16]. Even though contributing to more than 50% of the workforce in the medical field, women's under-representation in publication and research is still a significant matter of concern. Recent data from the Oculoplastic Association of India (2012-2018), Vitreo-Retinal Society of India (2001-2019), and the Young Ophthalmologists Society of India (2019) suggest gender disparity favouring males [10].

The All India Ophthalmological Society (AIOS), established in 1930, is a registered society with a steadily growing membership (currently over 23,669 life members; gender data unavailable) [17]. The AIOS included 26 state-affiliated societies and 19 affiliated ophthalmic (other than state) societies. This retrospective, observational study examined the gender distribution of AIOS awardees over the past 73 years. Gender differences were examined while accounting for award year and other variables that may impact gender disparities.

## Materials and methods

This observational study aimed to analyze the gender distribution of award recipients within the AIOS from 1949 to 2022, to identify any gender disparities over this extensive period. Ethical review was not required, as the study utilized publicly available data. The described research adhered to the tenets of the Declaration of Helsinki.

Data were sourced from the AIOS website, specifically from the awards section accessible through the member zone. The data collection involved navigating to this section and recording details on a pre-designed structured proforma.

For the purpose of this study, the awards were categorized into three main types. The achievement award recognized individuals who have made exceptional contributions to the field of ophthalmology at either the international or national level. The scientific investigation/research award is granted to those who have made significant clinical or research contributions that have advanced the field. Finally, the leadership/service to the society award honors individuals who have shown outstanding leadership in the ophthalmology field and have provided notable service, particularly through their contributions to the AIOS.

The awards analyzed in this study included several categories: general awards, best free paper award, specialty awards (for free papers (oral) read at the Annual All India Ophthalmological Conference (AIOC), Best Scientific Poster Award, AIOS - Physical Poster Award, AIOS - Poster Podium Presentation, and the Best Video Award. However, it was noted that complete data, including recipients' names, were not available for all categories. Specifically, data were only available for the General Awards, Best Free Paper Award, Specialty awards (for free paper oral), the Best Scientific Poster Award, and the Best Video Award. Consequently, only 31 award categories were included in the analysis. The recorded information was then managed using Microsoft Excel (Microsoft® Corp., Redmond, WA). Each recipient's gender was categorized as male or female based on the available information. Since the AIOS awards varied in periodicity, ranging from annual awards to those given once every four years, and eligibility required recipients to be AIOS members, only awards with available recipient data and consistent periodicity were included in the final analysis. The data were extracted manually from the IOS website to determine gender identity. Gender was determined by only names (not pronouns) and photographs of the awardees.

For data analysis, Statistical Product and Service Solutions (SPSS, version 22; IBM SPSS Statistics for Windows, Armonk, NY) was used. Descriptive statistics were used to describe baseline categorical data, which was expressed as frequency with percentage. For inferential statistics, the chi-square test and Fisher’s exact test were employed to assess associations between categorical variables where applicable.

## Results

Among the 36 award categories mentioned on the official website of the AIOS, 31 award categories had the details, including the names of the award recipients and eligibility criteria. From 1949 to 2022, a total of 452 recipients had received awards under different categories and sub-specialties. Of the 452 award recipients, 397 (88.2%) recipients' gender was determined by gender-specific nouns and pronouns. The gender of 54 (11.8%) awardees was determined through their photographs posted on social media and/or the AIOS website.

Awards given by the AIOS from 1949 to 2022 are shown in Table 1. Of the 30 named awards, only three were named after females.

**Table 1:** Name of the awards given by the All India Ophthalmological Society from 1949 to 2022. * Award named after a woman

Sr. no	Award name	Number of award recipients
1	P Siva Reddy International Award	36
2	AIOS – C N Shroff Award	10
3	P. Awasthi Award	03
4	AIOS – K R Dutta Award	08
5	AIOS - R. P. Dhanda Award	08
6	Adenwalla Award	18
7	E. V. Srinivasan Award	11
8	AIOS – S C Dutt Award	07
9	AIOS – B K Narayan Rao Award	09
10	AIOS – R N Mathur Award	03
11	Priti Natarajan Award*	02
12	Col. Rangachari Award	39
13	Sante Vision Award	20
14	J. S. Mahashabde Award	16
15	Apos K. Vengala Rao Award	04
16	Rema Mohan Award*	07
17	Apos Pradeep Swarup Award	04
18	K. C. Singhal Award 2021	07
19	AIOS – D B Chandra Disha Award	19
20	Apos Santosh Honavar Award	08
21	S. D. Athawale Award	20
22	Sujatha Savitri Rao Award*	07
23	Prem Prakash – Disha Award	16
24	Shiv Prasad Hardia Award	20
25	Rakesh Sharma Memorial Award	11
26	Narsing A. Rao Award	16
27	S. Natarajan Award	15
28	E. T. Selvam Award	25
29	C. S. Reshmi Award	33
30	Hanumantha Reddy Award 2018	08
31	AIOS Life Time Achievement award	42

Gender distribution

Between 1949 and 2022, there were a total of 452 award recipients in 31 distinct major award categories bestowed by the AIOS. Of these, 323 award recipients were men (71.5%) and 129 (28.5%) were women. Among the 70 recipients of the achievement category award, more than 3/4th of the gender representation was dominated by males. In the scientific investigation/research items category (322 recipients), men received 65% of the awards. Although men still dominated this category, the gender gap was less pronounced than in the achievement category award. In the contribution to the society category, a total of 60 awards were given; among them, men received 58 awards (96.7%), and women only two awards (3.3%), reflecting a marked under-representation of women in recognition for societal contributions within the ophthalmology field (Table 2).

**Table 2:** Gender distribution for award recipients in the All India Ophthalmological Society from 1949 to 2022. Chi-square tests were used with statistical significance set at a P-value of <0.05.

Awards	Men; No.(%)	Women; No.(%)	Test Statistical Value	P-value
All (n=452)	323 (71.5%)	129 (28.5%)		
Achievement (n=70)	55 (78.6%)	15 (21.4%)	26.6 (chi-square)	<0.01
Scientific investigation/Research items (n=322)	210 (65.2%)	112 (34.8%)		
Contribution to the Society/Lifetime Achievement (n=60)	58 (96.7%)	2 (3.3%)		

Factors influencing gender distribution

Gender distribution of awardees was assessed for factors such as year of award, age of the award recipient, nomination, and type of award. The year of the awards (before or after 2000), nomination and type of awards (lifetime achievement or others), and age criteria of the awards, significantly affected the gender distribution of recipients (Table 3).

**Table 3:** Factors influencing the gender distribution of award recipients in the All India Ophthalmological Society from 1949 to 2022. * p < 0.05 indicates statistical significance.

Category		Men; No. (%)	Women; No.(%)	Test statistical value	P-value
Year of award	Before the year 2000 (n=96)	83 (86.4)	13 (13.5%)	13.4 (chi-square)	<0.01*
	After the year 2000 (n=356)	240 (67.4%)	116 (32.5%)		
Award given for work done at the international/national level	National (n=416)	296 (71.15%)	120 (28.84%)	0.362 (chi-square)	0.362
	International/anywhere in the world (n=36)	27 (75%)	9 (25%)		
Age of awardees (based on the age criteria of each award)	>60 years (n=63)	61 (96.82%)	2 (3.28%)	24.3 (chi-square)	<0.01*
	<60 years (n=7)	6 (85.71%)	1 (14.28%)		
	Age no bar (n=382)	256 (67.19%	126 (33.07%)		
Nomination category of all awardees	Self (n=373)	249 (66.75%)	124 (33.27%)	<0.01 (Fisher’s exact test)	<0.01*
	Society (n=60)	58 (96.66%)	2 (3.33%)		

**Figure 1:**
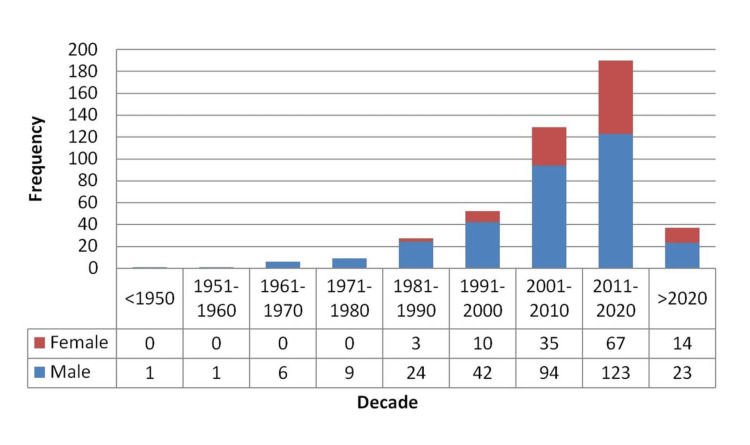
The trend of gender distribution of awardees over the decades.

Notably, female representation among awardees improved markedly in the last two decades, rising from just 13.5% before 2000 to 32.6% after 2000, a statistically significant change (p < 0.01) (Figure 1).

Gender distribution of awardees in different subspecialties of ophthalmology

**Figure 2:**
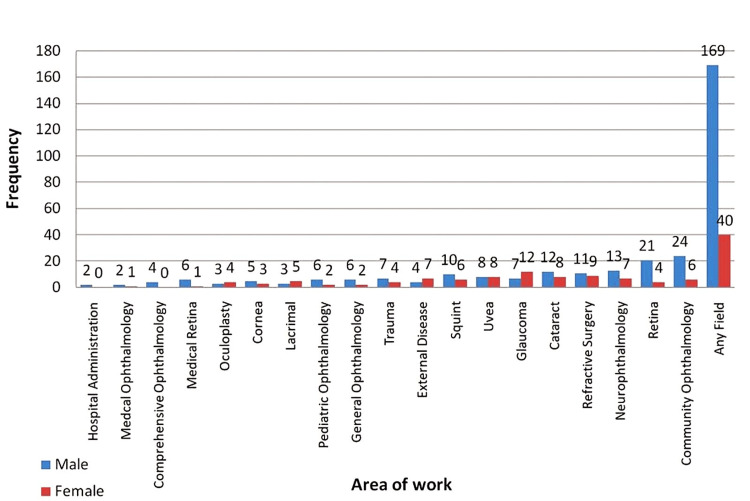
Area of work of the awardees in the All India Ophthalmological Society from 1949 to 2022.

In different subspecialties/the area of work in ophthalmology, hospital administration, and comprehensive ophthalmology fields showed 0% women representation, whereas oculoplastics, external disease, lacrimal diseases, and glaucoma showed > 50% women representation (57.14%, 72.8%, 62.50%, and 63.15%, respectively) (Figure 2). 

## Discussion

Despite significant progress in gender representation in medical schools and the growing visibility of women in academic medicine, gender disparity persists in healthcare across various dimensions [1,2,11,16]. The current study is the first to assess the gender disparity in one of the largest Indian ophthalmological societies. Overall, the study findings indicate a persistent gender disparity in award recognition within the AIOS, with men significantly outnumbering women across most award categories and timeframes. While there has been some improvement in female representation after the year 2000, significant challenges remain in achieving gender equity in the acknowledgment of contributions to the field of ophthalmology.

Women received awards (28.5%) at a lower rate than men (71.5%), coinciding with the results of previous studies from international ophthalmic societies [10,11,18]. The low female representation among awardees may be due to career breaks and relatively low research and publication productivity due to multidimensional roles in personal and professional life [19]. Other unsaid reasons might be implicit gender bias by the male counterparts, disparities in available funding, unequal opportunities, and inaccessible mentorship and sponsorship programs for females [12,20]. However, it is difficult to assess the actual contribution of each aspect to the evident disparity, considering that no previously published information exists on this sensitive subject.

Temporal trends indicate a significant male preponderance among award recipients before the year 2000, with a noticeable improvement in female representation after the year 2000. The proportion of women recipients increased from 13.5% before the year 2000 to 32.6% after the year 2000. The consistent improvement in women's representation (19.2%, 27.1%, and 35.6% in 1991-2000, 2001-2010, and 2011-2020 decades, respectively) depicts a positive trend and coincides with previously published results from most international ophthalmological societies [10,11]. This demonstrates the increased participation and recognition of women in academic spaces and increasing awareness of the importance of gender equity and balance [21].

Despite the positive trends, an observed significant gap, especially in major awards, such as lifetime achievement awards and awards achieved after society nomination (with minimal women representation; 3.3% and 3.3%, respectively), still deserves more objective introspection by academic committees for the criteria for award nomination. The present study results for achievement and scientific research categories awards coincide with the study by Nguyen et al. [11]. However, a much lower representation was observed for the “service to the society awards” in the current study [11]. Achievement and service to the society awards are likely the most significant awards, as these represent wholesome accomplishment in the field or a significant contribution to the AIOS. Contrastingly, the awards given for scientific investigation/research had a relatively better female representation (34.7%). This is in line with the increasing number of female residents opting for ophthalmology residency programs. Furthermore, representation varied greatly in subspecialty awards, with specialities other than external ocular diseases (including glaucoma, lacrimal, and oculoplastics) being dominated by male award recipients.

Furthermore, a significant gender gap was observed in categories with age as an eligibility criterion. This coincides with other studies demonstrating a significant gender gap in high academic positions, including an online survey study with only 7.4% of 529 Indian women ophthalmologists holding the position of head of their respective departments [19]. The relatively lower representation in higher ranks in the older age group suggests the time trend in gender disparity evident in this study. These findings are in unison with other surgical society awards, where women often receive more diversity-focused awards rather than leadership awards [22].

Award selection in a society is often a subtle, indirect, and multistep process that includes nomination of a candidate, review of their work or contribution, and final selection by the awarding committee. At every step, there are clear opportunities to implement measures that can help mitigate bias and increase equity [23]. To ensure diversity and equity, societies should standardise award selection and assessment criteria by anonymising the names and genders of candidates. As an initial acknowledgment of the issues, the availability of gender membership data and the society's statement on gender equity and diversity on its website are important initial steps to strengthen the faith and avoid systematic biases. Creation of accessible structured mentorship programs, fostering sponsorship opportunities, and providing support networks would further empower women to reach their full potential, paving the way for a more diverse, inclusive, and equitable profession [24,25].

To the best of our knowledge, this is the first study to widely examine the female representation among awardees of India's largest ophthalmic society, which is the benchmark for most state and non-state societies. However, this study is not short of limitations. Many awards had small denominators (<25) to confidently reflect the accurate proportion. AIOS-registered members' gender data were not available publicly. Hence, its correlation with award recipients could not be done. The data source was the AIOS website, which was subject to problems such as incomplete or erroneous information. Due to manual extraction of data and determination of gender identity by only name (not pronouns) and photographs of the awardees, nonbinary identities could have been missed in the present study.

## Conclusions

This study indicates an improvement in female representation among the award recipients in the AIOS over the past few decades. However, marked underrepresentation in the major prestigious awards, such as the lifetime achievement awards, is disheartening. Although the reasons may be manifold, it is important to systematically establish an investigation into the myriad of potential factors that might hinder women from advancing in the ophthalmology field. Future studies across different medical and surgical societies (including ophthalmology societies) across India can help identify reasons for gender equity and reduce the gender gap.
